# Stratifying Intraductal Papillary Mucinous Neoplasms by Cyst Fluid Analysis: Present and Future

**DOI:** 10.3390/ijms21031147

**Published:** 2020-02-09

**Authors:** Scarlett Hao, Caitlin Takahashi, Rebecca A. Snyder, Alexander A. Parikh

**Affiliations:** 1 Department of Surgery, Brody School of Medicine at East Carolina University, Greenville, NC 27834, USA; haos17@ecu.edu (S.H.); pipkinca19@ecu.edu (C.T.); 2 Division of Surgical Oncology, Department of Surgery, Brody School of Medicine at East Carolina University, Greenville, NC 27834, USA; snyderre19@ecu.edu

**Keywords:** intraductal papillary mucinous neoplasms, cyst fluid analysis, genetic changes, genomics/proteomics/lipidomics

## Abstract

A significant proportion of patients with intraductal papillary mucinous neoplasms (IPMNs) undergo surgical resection in order to prevent or treat pancreatic cancer at the risk of significant perioperative morbidity. Efforts have been made to stratify the potential risk of malignancy based on the clinical and radiographic features of IPMN to delineate which cysts warrant resection versus observation. An analysis of the cyst fluid obtained by preoperative endoscopic examination appears to be correlative of cyst type and risk, whereas serum markers and radiographic findings have not yet reached a level of sensitivity or specificity that proves they are clinically meaningful. In this review, we investigate the current cyst fluid analysis studies and present those that have shown promise in effectively stratifying high-risk versus low-risk lesions. While new cyst fluid markers continue to be identified, additional efforts in testing panels and marker composites in conjunction with clinical algorithms have also shown promise in distinguishing dysplasia and the risk of malignancy. These should be tested prospectively in order to determine their role in guiding the surveillance of low-risk lesions and to evaluate the new markers detected by proteomics and genetic sequencing.

## 1. Introduction

The incidence of intraductal papillary mucinous neoplasms (IPMNs) has increased significantly with the development and increasing use of cross-sectional non-invasive imaging, such as computed tomography (CT), magnetic resonance imaging (MRI), and magnetic resonance cholangiopancreatography (MRCP). These imaging modalities will reveal an incidental pancreatic lesion in up to 45% of patients [1,2,3,4]. Many incidentally identified pancreatic cysts are IPMNs, a subset of which may be precursors of pancreatic adenocarcinoma (PDAC) [5,6,7].

PDAC is a devastating diagnosis with a <10% 5-year overall survival rate for all comers, and unfortunately no method of screening for pancreatic cancer has been proven to be either effective or practical [8]. Similar to colorectal and other cancers, the resection of a premalignant lesion such as IPMN is thought to improve survival. However, the unknown natural history of IPMN malignant degeneration into PDAC, as well as the significant morbidity of a pancreatic resection, has created controversy over which patients should undergo surgical resection. Therefore, the management for IPMNs remains controversial [9,10].

The initial Sendai guidelines for IPMN management were published in 2006, updated to the Fukuoka guidelines published in 2012, and were updated again in 2017 [11,12,13]. These guidelines (otherwise known as the International Consensus Guidelines or ICG) characterize IPMNs as main-duct IPMNs (MD-IPMN), branch-duct IPMNs (BD-IPMN), and mixed-type IPMNs based on imaging and histology. MD-IPMNs are defined as having a main duct dilation >5 mm without other identifiable reasons for obstruction. BD-IPMNs are cysts >5 mm that communicate with the pancreatic duct. The mixed type meet the criteria for both BD- and MD-IPMN.

These guidelines define three categories of all IPMNs; those considered low-risk, those with “worrisome features,” and those with “high-risk stigmata.” “Worrisome features” include a cyst size ≥3 cm, an enhancing mural nodule <5 mm, thickened enhanced cyst walls, a main pancreatic duct (MPD) size in the range 5–9 mm, an abrupt change in the MPD, lymphadenopathy, elevated CA 19-9, and a rapid cyst growth rate (more than 5 mm over 2 years). “High-risk stigmata” include obstructive jaundice with cystic lesions in the pancreatic head, an enhanced mural nodule ≥5 mm, and an MPD ≥10 mm.

The Fukuoka guidelines recommend the resection of BD- and MD-IPMNs with “high-risk stigmata” due to the increased risk of underlying malignancy. For BD-IPMNs, absolute indications for resection also include cytology positive for high grade dysplasia. IPMNs with “worrisome features” and endoscopic ultrasound (EUS) with negative findings should have imaging and EUS surveillance based on cyst size [13]. In secondary analyses, the specificity of the Fukuoka guidelines reaches 94%–97% in some studies, with sensitivity only reaching 28%–62% [14,15]

The American Gastroenterological Association (AGA) guidelines for pancreatic cysts also define “high-risk” lesion characteristics as a size ≥3 cm, a dilated MPD, and mural nodules [16]. For high risk lesions, EUS with a fine needle aspiration (FNA) of cyst fluid is recommended for further analysis, which then informs the decision to observe or resect. These guidelines have demonstrated improved sensitivity (50%) and accuracy (73.7%) compared to the Fukuoka guidelines but are less specific (89.1%) for the management of pancreatic cysts [16].

Although the specificity of these criteria are improving, there continues to be a significant percentage of patients who undergo pancreatic resection for noninvasive lesions; up to 77% may be benign on postoperative pathological examination [7,17,18,19,20]. Additionally, the available data is based on retrospective studies of surgical patients, so the true percentage of invasive or high-risk lesions is even lower when including patients not selected for resection. Given the risk of surgical morbidity, this calls for further research into more objective tests to better identify patients with IPMN that are at the greatest risk of malignancy, namely IPMN with high-grade dysplasia (HGD) or patients associated with an invasive component. Furthermore, even in patients with HGD, the risk of progressing to an invasive malignancy has not been well defined. Current research has focused on tissue genetics, tumor markers, and cyst fluid analysis with promising results [13]. Utilizing EUS–FNA to obtain cyst fluid samples for analysis in the pre-operative setting has demonstrated significant potential for diagnosis and the guidance of surgical management without incurring additional risks of peritoneal seeding [21,22]. Here, we present an updated review on the most promising cyst fluid markers in order to summarize current strategies for risk stratification and to identify future directions for further investigation.

## 2. Single Marker Analyses

Cytology studies on EUS-obtained cyst fluid samples have historically been of great importance in the assessment of the malignancy risk of IPMN. However, the high sensitivity and specificity of cytologic studies have been impeded by multiple factors, including interobserver variability, low cellular yield, low volume yield, and even, when there is enough of a sample for cytologic studies, the percent of “indeterminate” or “nondiagnostic” results can reach greater than 40% [23,24]. Efforts to improve the diagnostic yield of cyst fluid samples include additional techniques such as Moray micro forceps biopsy [25]. While 0.4 mL is needed for DNA analysis, up to 1.0 mL is needed for carcinoembryonic antigen (CEA) analysis and more if seeking to perform cytology and send for sequencing studies [24,26,27].

Given the limitations of cytology, the search for quantifiable cyst fluid biomarkers began with proteins and classically known tumor markers. Amylase in the cyst fluid would be consistent with ductal communication, and levels <250 U/L indicated lack of ductal communication with a specificity of 98% [28,29]. Low amylase, however, does not rule out malignancy [30]. CAE is an epithelial glycoprotein suggestive of mucin production [28]. Elevated levels of CEA > 192 ng/mL in combination with amylase in cyst fluid have been shown to identify mucinous cysts with a sensitivity of 64%–78% and a specificity of 65%–89% (Table 1) [26,28,31,32,33]. However, these two markers do not discriminate between levels of dysplasia in IPMN and do not reliably distinguish between IPMN and mucinous cystic neoplasms (MCN); some MCNs also have elevated amylase [32,34].

Other genetic mutations have also been shown to distinguish between mucinous and non-mucinous cysts and their subtypes, including serous cystadenoma (SCN) and solid-pseudopapillary neoplasm (SPN) (Table 2). The guanine-nucleotide-binding protein-alpha stimulating (GNAS) and the Kirsten rat sarcoma viral oncogene homolog (KRAS), an oncogene and proto-oncogene, respectively, have also been identified as commonly mutated in pancreatic cyst fluid [26,35]. GNAS in particular has been shown to distinguish IPMN from MCN. Wu et al. demonstrated 98% sensitivity and 100% specificity in diagnosing IPMN when testing cyst fluid [35]. Other single markers, such as glucose and neutrophil gelatinase-associated lipocalin (NGAL), have also been investigated with no ability to stratify IPMN [36,37,38]. Fortunately, there are now several single markers with the ability to both identify and grade IPMN dysplasia.

### 2.1. Mucins

Mucins (MUC) are highly glycosylated proteins released into the cyst from the epithelium, and their subtypes vary with the histopathological IPMN subtypes: gastric, intestinal, pancreatobiliary, and oncocytic [39,40]. Given that IPMN subtypes also correlate with specific degrees of dysplasia, Maker et al. investigated the ability of mucin to determine the degree of IPMN dysplasia [41]. In this study, investigators demonstrated a significant increase in MUC2 and MUC4 among cysts with HGD and increased MUC2 in intestinal type IPMNs, which have a higher degree of malignant transformation [41]. A more recent study demonstrated that mucins can carry abnormal glycoforms, finding that oligosaccharide linkages *α*GlcNAc and *β*GlcNAc associated with MUC5AC were associated with malignancy. The version of MUC5AC with *α*GlcNAc was found primarily in IPMN cyst fluid compared to MCN cyst fluid, and the level of staining on final pathologic specimens increased with degree of dysplasia [42]. However, the study did not show if cyst fluid levels also varied with degree of dysplasia, limiting its preoperative diagnostic utility.

### 2.2. IL-1b

Cytokine expression within pancreatic cyst fluid has also been shown to be prognostic. Higher concentrations of interleukins (IL-) 1b, 5, and 8 have been identified in cysts containing HGD or malignancy (*p* < 0.0001) [43]. By multivariate analysis, IL-1b was found to be an independent factor in predicting high-risk versus low-risk pancreatic cysts with a positive predictive value of 71% and a negative predictive value of 75%, as well as sensitivity and specificity reaching 79% and 95% [43]. Cyst fluid IL-1b remains a prime target among the pool of cytokines that otherwise did not correlate or had very low expression levels.

### 2.3. PGE2

Prostaglandin E2 (PGE2) levels have been previously shown to be elevated in pancreatic cancer tissue, prompting investigations into its utility in diagnosing premalignant pancreatic cysts. Schmidt et al. prospectively studied cyst fluid samples from 65 patients with pancreatic cystic neoplasms [44]. Using enzyme-linked immunosorbent assays (ELISA), they quantified the concentration of PGE2 and found higher levels of PGE2 in IPMNs compared to MCNs (*p* < 0.05) and demonstrated that PGE2 concentration correlated stepwise with the degree of dysplasia within an IPMN. It was noted that PGE2 concentrations were also higher amongst patients who had a PDAC not associated with the coexisting IPMN [44]. Their results were subsequently replicated within a larger cohort of 100 patients with similar results. On multivariable analysis, PGE2 alone was significantly associated with HGD-IPMN dysplasia with a sensitivity of 63% and a specificity of 79% [45].

### 2.4. Telomere Fusion Status

Telomere shortening and fusion have been identified in pancreatic malignant degeneration due to chromosomal instability. IPMNs with associated dysplasia have been shown to carry shortened chromosomal telomeres [46]. Hata et al. were able to demonstrate telomere fusion in 0% of IPMNs with low-grade dysplasia (LGD) and increasing copy numbers with HGD-IPMN and IPMN with adenocarcinoma [47]. In some patients, there were fusions detected within IPMNs after histological interpretation, but not initially in cyst fluid analysis. This is a limitation of using telomere fusion as a preoperative diagnostic tool because it depends on the shedding of DNA into the cyst fluid, which may be uncommon [47].

### 2.5. miR-216a

MicroRNA (miRNA) profiling using Next Generation Sequencing (NGS) is a newer area of cancer research, with demonstrable aberrant miRNA expression in pancreatic cancer and pancreatic cysts [48,49,50]. Wang et al. sought to investigate the so-called ”miRNome” of IPMN cyst fluid [50]. Of the 15 miRNAs investigated, miR-216 was the most strongly associated with dysplasia, with a higher expression of miR-216 in HGD-IPMN and IPMNs with adenocarcinoma compared to LGD IPMN (*p* = 0.011 and *p* = 0.020). Although, there were no statistical differences between HGD and adenocarcinoma (*p* = 0.540) [50]. MicroRNA has, thus far, demonstrated significant potential in stratifying IPMNs.

### 2.6. CEP and mAb Das-1

The murine Das-1 monoclonal antibody (mAb) was created to react with a normal colon epithelial protein (CEP), based on the observation that these cell types are not normally present in gastric and pancreatic epithelium and are prone to developing invasive carcinoma when present [51]. This unique immunoreactivity has been demonstrated on resected pancreas specimens with HGD-IPMN and PDAC, leading to the most recent study evaluating its applicability to preoperatively sampled cyst fluid [52]. Das et al. investigated 169 patients with pancreatic cystic lesions across 4 institutions and found that non-mucinous and low-risk cysts displayed little reactivity, whereas HGD-IPMN and MCN lesions had significantly higher reactivity (*p* < 0.001), with a sensitivity of 88.3% and a specificity of 92.7% when stratifying for HGD or invasive malignancy [52]. Based on their internal comparative evaluation, Das-1 reactivity has significant potential in distinguishing HGD or malignancy.

## 3. Panel Analyses

The exact pathophysiologic progression of IPMN towards frank adenocarcinoma is still unknown, but the pathology of resected specimens has given insight into the degeneration of the epithelial lining into further dysplasia [40]. This change in cell dysplasia is thought to give rise to a specific environmental milieu deposited into the cyst fluid [43]. Since no single marker has thus far demonstrated strong predictive power, there are ongoing efforts now to investigate panels or combinations of several markers in hopes of better identifying high-risk versus low-risk IPMNs.

### 3.1. Mutational Analysis: KRAS, GNAS, and Cytopathology

Despite the individual limitations of sampling for KRAS or GNAS to preoperatively assess IPMN malignancy risk, Bournet et al. sought to evaluate the predictive power of a combinatorial assay examining KRAS, GNAS, and cyst fluid cytopathology [53]. Cyst fluid samples were evaluated for specific codon mutations using custom DNA probes and polymerase chain reaction (PCR) amplification. The triple combination predicted malignant IPMN with an excellent sensitivity of 92% but a limited specificity of only 50% [53]. Furthermore, a mutational analysis was performed on a small cohort using Sanger sequencing to detect any KRAS and GNAS mutations but failed to show a diagnostic advantage even when combined with cytopathology [54].

### 3.2. Next-Generation Sequencing Panel: KRAS, GNAS, TP53, PIK3CA, and PTEN

Further analysis of GNAS mutations and the addition of GNAS analysis to KRAS mutation findings remain controversial in pancreatic cyst diagnosis. There is evidence that GNAS mutations and KRAS mutations increase the sensitivity and specificity of diagnosing IPMNs to 98% and 84%, respectively, compared to single mutational analysis alone [55]. Singhi et al. attempted to design an NGS panel of the most frequent and well-known mutations associated with pancreatic cancer, including BRAF, NRAS, TP53, and PIK3CA [56]. Their best sensitivity and specificity to detect IPMNs with advanced neoplasia (defined by the presence of HGD or an invasive component) was achieved with a composite of KRAS and/or GNAS with TP53, PIK3CA, and/or PTEN (88% and 97%), which is now part of a commercially available panel dubbed PancreaSeq [57]. These values increased further if the mutant allele frequencies were set at a specific threshold [56]. Where Sanger sequencing had previously failed to demonstrate utility for combination panels that included KRAS/GNAS, the lower limit of detection available using NGS has allowed a useful revisit of the classic gene mutations.

### 3.3. Whole Exome Sequencing: SMAD4, RNF43, Chromosomal Aneuploidy, and TP53

Based on prior whole exome sequencing to profile pancreatic cysts, Springer et al. performed whole exome sequencing on cyst fluid samples to evaluate specific low frequency allele mutations in a sample of 11 genes (BRAF, CDKN2A, CTNNB1, GNAS, KRAS, NRAS, PIK3CA, RNF43, SMAD4, TP53, and VHL) as well as the loss of heterozygosity (LOH) and aneuploidy [58]. The sequenced data was then fed through an algorithm named Multivariate Organization of Combinatorial Alterations (MOCA), which tested and compared possible collections of markers for the best predictive composite set of markers. The most predictive composite occurred with SMAD4, RNF43, aneuploidy in either of the 4 chromosomal arms, and TP53. These 4 characteristics together predicted cysts that were appropriately resected (MCN, HGD-IPMN, IPMN with adenocarcinoma, and SPN) with a sensitivity of 75% and a specificity of 92% [58]. Separate composites were identified as well that effectively predicted the type of cyst including MCNs and SCNs. It is worth noting that sequencing was able to be performed successfully with just 0.25 mL of cyst fluid; however, the bulk of the study was performed on cyst fluid obtained at the time of surgical resection as opposed to preoperative EUS.

### 3.4. Proteomic Array: MMP9, CA72-4, sFASL, and IL-4

Where prior studies have cross-validated markers on the initial patient data sets that generated the markers, a group at Memorial Sloan Kettering has applied their predictive models to an independent multi-institutional set of patients [59]. In this study, investigators identified 4 markers based on a proteomics antibody bead array with a strong predictive value for high-risk lesions (HGD-IPMN and invasive IPMN). The 2 sets of markers (MMP9 and CA 72-4, sFASL and IL-4) were evaluated in the cyst fluid samples of 149 patients across 3 high volume institutions, with reported concordance indices of 0.76 and 0.8, respectively. While the samples were obtained at the time of surgical resection, the data still suggests high value in further investigating protein expression in cyst fluid as a means of stratifying lesions preoperatively.

### 3.5. Biosignatures

In the context of contemporary data on markers in the pancreatic cyst genome, proteome, miRnome, and metabolome, Maker et al. assembled a comprehensive assay to test the gene expression of all of the most predictive markers to discover the genetic “biosignature” of high-risk IPMNs [60]. Cyst fluid samples obtained from several centers across the United States and Europe were run through the single sequencing panel, testing for IL-1b, muc-1, muc-2, muc-4, muc-5ac, muc-7, PTER2, PTGS1, prostaglandin E2-R, KRAS, GNAS, GAPDH, RPLP0, TP63, ERBB2, and prostaglandin E synthase 2 as well as 16 individual miRNAs. The quantification data was then analyzed for the most optimal set requiring the least number of markers to correctly stratify low- and high-risk IPMN. The most predictive signature was found to be a combination of IL-1B, muc-4, and prostaglandin E synthase with either a mutation in GNAS or KRAS, with a concordance index of 0.86 [60]. The model was built on cyst fluid obtained from pathologically confirmed high-risk lesions, so its predictive value remains to be tested on a prospective cohort.

## 4. Nomograms and Algorithms: Combining Clinical Features and Biomarker Analyses

While published guidelines direct decision making based on specific clinical, imaging, and laboratory factors, the investigation of the features that are most predictive of malignancy continues, especially given the numbers of patients who undergo surgical resection based on guideline-directed decision making who ultimately have benign pathology. As we have outlined, existing clinical, imaging, and laboratory markers found to be associated with malignancy have been studied in various combinations in hopes of achieving increased predictive statistical power greater than achievable with a single feature. These have also been combined with biomarker cyst fluid analyses in an attempt to reach even greater sensitivity and specificity.

### 4.1. Nomogram

The clinical and imaging details of over 1000 patients across three institutions were utilized in a study published in 2018 to generate various multivariate models [61]. The models generated closely mirror the same parameters described in the Fukuoka guidelines, including the mural nodule and duct diameter, and were assembled into nomograms with a point system correlating with the probability of high-risk IPMN. Unfortunately, laboratory values such as Ca 19-9 were not available for a significant proportion of patients and were therefore excluded from the multivariable modeling. Additionally, the nomogram only provides a probability of high-risk on a continuous scale for a given patient’s IPMN, serving as another adjunctive piece of information to consider rather than a definitive diagnostic test.

**Figure 1:**
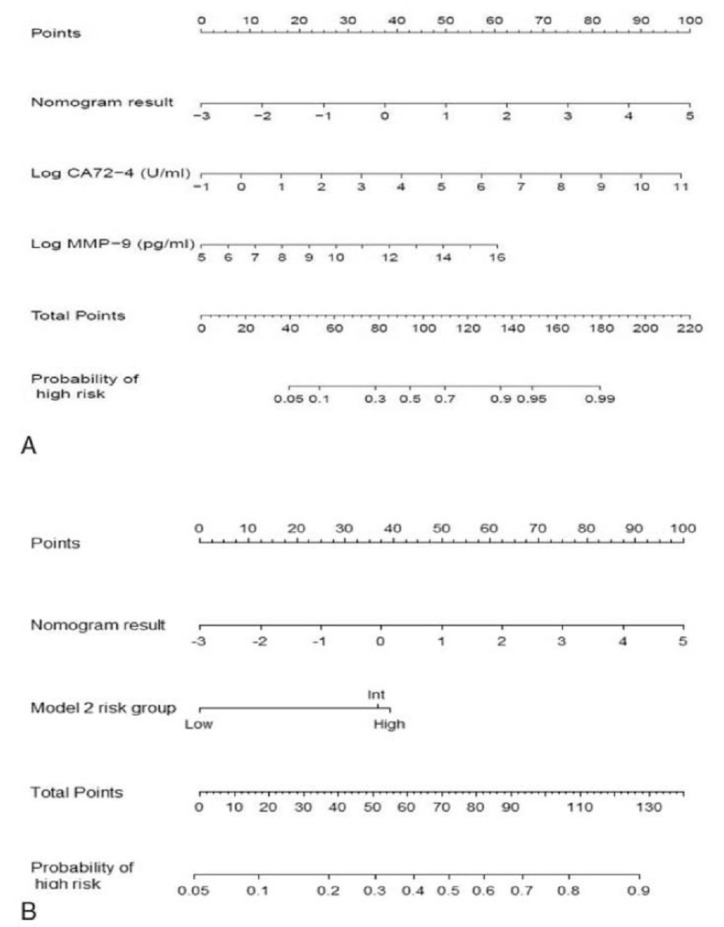
Clinical factors and cyst fluid analysis combined into a single nomogram for each model, based on factors MMP9 and CA 72-4 (part **A**), and sFASL and IL-4 (part **B**) [59]. This data was reprinted under the Creative Commons License.

With the growing body of literature on highly correlative biomarkers identified in cyst fluid analysis, the same research group sought to combine their nomogram with their proteomic biomarker models MMP9 and CA 72-4, sFASL, and IL-4 (Figure 1). The combination of their two biomarker models with the nomogram improves concordance indices up to 0.88 [59].

### 4.2. MOCA Algorithm

In considering the need to include both “positive” and “negative” features in order to maximize sensitivity and specificity as well as to efficiently handle the large number of possible combinations, Masica et al. utilized the MOCA application to simultaneously generate, test, and verify combinations of a given set of features [62]. The MOCA application was originally developed to identify correlating features in drug responses and mutations in the progression of malignancy [63,64]. Not unsurprisingly, the top group of parameters included cyst size, ductal dilation, and the presence of jaundice, with a sensitivity and specificity of 81% and 61%, respectively [62].

As the authors state that the addition of biomarkers could potentially further improve the statistical power of their model [62]. In the previously described study published by Springer et al., the resultant composite of SMAD4, RNF43, chromosomal aneuploidy, and TP53 were subsequently combined with the MOCA-identified composite of clinical features with an ultimate sensitivity and specificity of 75% and 92%, respectively, in identifying pancreatic cysts requiring surgery, outperforming the sensitivity of the Fukuoka guidelines but at the cost of specificity [58].

### 4.3. IMP Testing

Over the past decade, another modality of integrating clinical features and cyst fluid analyses to assess the malignancy risk of pancreatic cysts was developed from the growing body of literature. Interpace Diagnostics (Parsippany, NJ) has offered PancraGEN, a propietary integrated molecular pathology (IMP) test that generates an aggregate malignancy risk score based on the highly correlative clinical features (cyst size, duct diameter, etc) in addition to cyst fluid analysis testing for LOH, the quality of DNA, and genetic mutations using Sanger sequencing [65]. Simpson et al. evaluated prospectively collected IPMN patients that had undergone IMP testing, including patients deemed to have a low risk IPMN who were applied to an institution specific surveillance protocol [66]. In addition, given the limited specificity, the same investigators critically analyzed the clinical features portion of the IMP and assessed a newly dubbed IMP-10, where the only clinical feature combined with the cyst fluid analyses was duct diameter [67]. While IMP testing remained highly sensitive (up to 88.9%), IMP-10 enabled a high specificity (up to 90.1%) for invasive IPMN or PDAC. Both tests had decreased sensitivity and specificity when used to predict invasive pathology or HGD-IPMN. It is worth noting that the patients who underwent IMP testing specifically had a negative cytology, so the authors concluded that IMP testing did have a benefit in determining malignant and invasive risks in the setting of a negative cyst fluid cytology [67].

## 5. Future Directions

**Figure 2:**
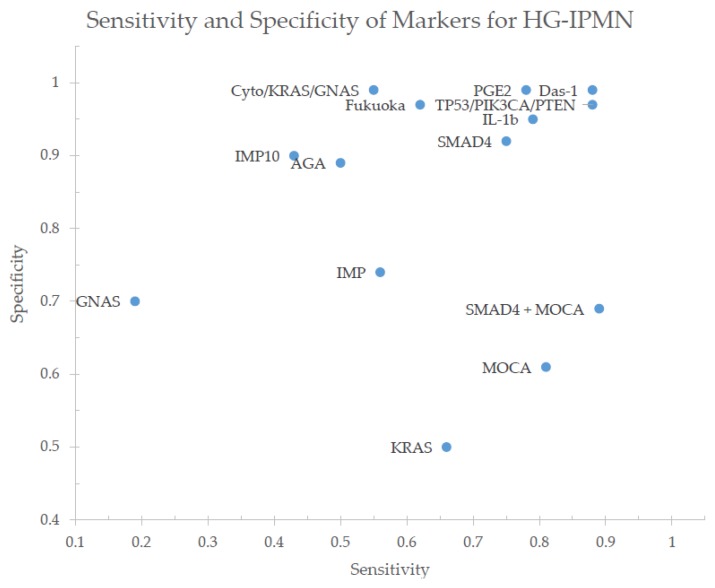
Sensitivities and specificities of the single-marker and composite-marker analyses identifying high-grade and invasive intraductal papillary mucinous neoplasm (IPMN) as provided by the discussed studies [13,16,43,44,52,53,56,62,66,67].

The state of current biomarker analyses continues to progress towards higher diagnostic performance, with sensitivities and specificities outperforming published guidelines (Figure 2), however, their current use remains as an adjunct to the decision-making process [68]. Existing studies have been limited by (1) the decision to take patients to surgery based on other factors excluding the cyst fluid analysis, (2) inadequate cyst fluid samples for analysis, (3) limited follow-up for patients who do not undergo surgical resection, (4) assessment of the test accuracy retrospectively, although specimens may be collected prospectively [58,67,69]. Given the improved performance of these cyst fluid analyses, should they be given greater weight in deciding how to proceed? Will there be a reduction in the rate of benign specimens without compromising the false negative rate (i.e., not resecting an invasive lesion)? Additionally, a subset of the studies is based on cyst fluid obtained postoperatively. There are many other limitations, and validation studies are needed for many of the above biomarkers. Thereafter, prospective clinical trials with longitudinal follow-up and outcome tracking are warranted.

Cyst fluid analyses may also have a role in surveillance. If a cyst is deemed to have a low enough risk to surveil safely rather than resect, current guidelines recommend radiographic surveillance. Clearly, there is a subset of invasive lesions that do not have characteristic radiographic findings or meet the guideline criteria [69]. However, the uncertain natural history of IPMN malignant degeneration makes the timing to repeat cyst fluid sampling and analysis equally uncertain.

Fortunately, there are ongoing efforts to fund investigations to solve these unanswered questions, powered through the Pancreatic Cancer Action Network (PANCAN) and National Cancer Institute grants [70]. Several grants have already been awarded to exploit and study biomarkers for the early detection of malignancy and precursor lesions. In addition, although there are several promising single and composite biomarker assays, there remain many new and untested markers [50,71,72,73]. The critical role of biomarker analyses in the future of diagnosing and surveilling precursor pancreatic cysts is undeniable. The question of how it will be best utilized remains to be discovered.

## Figures and Tables

**Table 1:** Cyst type differentiation by carcinoembryonic antigen (CEA) and amylase levels [29,32].

	Amylase < 250	Amylase > 250
**CEA < 192**	SCA	Pseudocyst
**CEA > 192**	MCN	IPMN

**Table 2:** Genetic mutation profile of mucinous and non-mucinous cyst subtypes [34,36,37].

	IPMN	MCN	SCA	SPN
KRAS	+	+	−	−
GNAS	+	−	−	−
VHL	−	−	+	−
CTNNB1	−	−	−	+
SMAD4	+/−	+/−	−	−
RNF43	+	+	−	−
NGAL	−	−	−	−
glucose	−	−	+	+

## Abbreviations

IPMN	Intraductal papillary mucinous neoplasm
CT	Computed tomography
MRI	Magnetic resonance imaging
MRCP	Magnetic resonance cholangiopancreatography
PDAC	Pancreatic ductal adenocarcinoma
ICG	International consensus guidelines
MD-IPMN	Main duct IPMN
BD-IPMN	Branch duct IPMN
MPD	Main pancreatic duct
AGA	American Gastroenterological Association
EUS	Endoscopic ultrasound
FNA	Fine needle aspiration
HGD	High-grade dysplasia
CEA	Carcinoembryonic antigen
SCA	Serous cystadenoma
SPN	Solid pseudo-papillary neoplasm
MCN	Mucinous cystic neoplasm
GNAS	Guanine nucleotide binding protein, alpha-stimulating
KRAS	Kirsten rat sarcoma viral oncogene homolog
NGAL	neutrophil gelatinase-associated lipocalin
MUC	Mucin
IL	Interleukin
PGE2	Prostaglandin E2
ELISA	Enzyme-linked immunosorbent assay
NSAID	Nonsteroidal anti-inflammatory drug
LGD	Low-grade dysplasia
miRNA	MicroRNA
NGS	Next Generation Sequencing
mAb	Monoclonal antibody
CEP	Colon epithelial protein
PCR	Polymerase chain reaction
LOH	Loss of heterozygosity
MOCA	Multivariate organization of combinatorial alterations
IMP	Integrated molecular pathology
PANCAN	Pancreatic Cancer Action Network
NCI	National Cancer Institute
